# A review of the optimization of thyroid function, thrombophilia, immunity and uterine milieu: OPTIMUM treatment strategy for recurrent implantation failure and recurrent pregnancy loss

**DOI:** 10.1002/rmb2.12561

**Published:** 2024-01-18

**Authors:** Keiji Kuroda

**Affiliations:** ^1^ Center for Reproductive Medicine and Endoscopy Sugiyama Clinic Marunouchi Tokyo Japan; ^2^ Department of Obstetrics and Gynaecology Juntendo University Faculty of Medicine Tokyo Japan

**Keywords:** infertility, IVF, miscarriage, recurrent implantation failure, recurrent pregnancy loss

## Abstract

**Background:**

Aside from embryonic factors, various factors can intricately interfere with embryo implantation and maintenance of pregnancy, causing recurrent implantation failure (RIF) or recurrent pregnancy loss (RPL). This review focuses the optimization of thyroid function, thrombophilia, immunity, and uterine milieu (OPTIMUM) treatment strategy on RIF and RPL.

**Methods:**

Three studies employing the OPTIMUM treatment strategy for patients with RIF and/or RPL were reviewed.

**Results:**

The OPTIMUM improved pregnancy rates in women with RIF aged <40 years. Among advanced age women, however, no significant differences in pregnancy rates were observed between the control, OPTIMUM, and preimplantation genetic testing for aneuploidy (PGT‐A) groups, although pregnancy rates were highest after OPTIMUM + PGT‐A. The OPTIMUM reduced miscarriage rates in women with RPL aged <40 years. Among advanced age women, PGT‐A, but not the OPTIMUM, contributed to miscarriage prevention. Factors predicting pregnancy success in women with RIF who received the OPTIMUM included thrombophilia and young age. Risk factors for an unsuccessful live birth among women with RPL who received the OPTIMUM included advanced age, infertility, diminished ovarian reserve, and non‐ART treatment.

**Conclusions:**

The OPTIMUM can improve pregnancy outcomes in women with RIF/RPL, except for advanced age women with embryonic factor‐induced reproductive failure.

## Graphical abstract

This review focuses three studies of the optimization of thyroid function, thrombophilia, immunity, and uterine milieu (OPTIMUM) treatment strategy on recurrent implantation failure (RIF) or recurrent pregnancy loss (RPL). The OPTIMUM can improve pregnancy outcomes in women with RIF/RPL, except for advanced age women with embryonic factor‐induced reproductive failure.

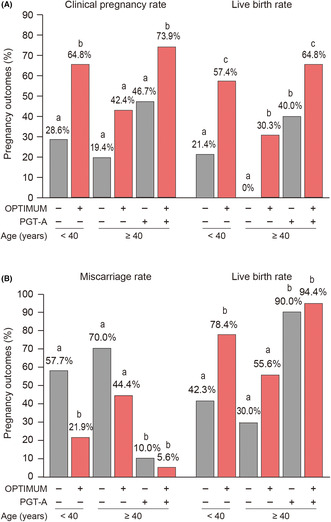

## 1 INTRODUCTION

The incidence of embryo wastage among humans is exceedingly high, with estimates showing rates as high as 30% prior to implantation, a further 30% before 6 weeks of gestation, and 10%–15% of all clinical pregnancies.^1^ Human embryos and fetuses with chromosomal abnormalities are excluded from the natural selection mechanism during the pregnancy process^2^, ^3^, ^4^; therefore, implantation failures and pregnancy losses are mainly caused by embryonic chromosomal abnormalities.^5^, ^6^ In line with this, women with a history of recurrent implantation failure (RIF) and recurrent pregnancy loss (RPL) may fortuitously suffer repeated sporadic reproductive failures. However, aside from embryonic factors, various other factors, including immune, endocrine, microbial, anatomic, hematologic, genetic, and lifestyle factors, have also been found to intricately interfere with the processes of embryo implantation and maintenance of pregnancy, causing multiple reproductive failures.^7^, ^8^, ^9^, ^10^, ^11^

In recent years, guidelines for the management of RIF and RPL have been published after evidence for each examination and treatment had been reviewed.^12^, ^13^, ^14^, ^15^ Despite the a lack of consensus on the optimum tests and treatment approaches, combination treatment based on diagnostic results from selected examinations is required for RIF and RPL, which have been considered multifactorial diseases. Our group had been the first to report on the optimization of thyroid function, thrombophilia, immunity, and uterine milieu (OPTIMUM) treatment strategy, which simply involves the detection of risk factors for implantation failure and pregnancy loss using minimum and inexpensive tests and treatment of the identified factors for the management of RIF.^16^ The OPTIMUM had been introduced as a treatment approached based on diagnostic findings in the European Society of Human Reproduction and Embryology (ESHRE) guideline for RIF.^15^ In addition, given the overlapping risk factors for implantation failure and pregnancy loss, one study also found that the OPTIMUM was effective for RPL.^17^ The OPTIMUM, which is the world's first treatment protocol for both RIF and RPL, has allowed most young women with a history of RIF and/or RPL to raise children successfully.^16^, ^17^ However, clinical studies on RIF and RPL have shown that the OPTIMUM did not significantly improve pregnancy outcomes among advanced age women.^16^, ^17^ Therefore, we also reported the outcomes of the OPTIMUM in combination with single euploid blastocyst transfer after preimplantation genetic testing for aneuploidy (PGT‐A) as a protocol developed for women aged 40 years or more.^18^ This review aimed to summarize the treatment procedures and indications of the OPTIMUM and to clarify its therapeutic effects on pregnancy outcomes in women who suffer from multiple reproductive failures.

## 2 PROTOCOL OF THE OPTIMUM TREATMENT STRATEGY

### 2.1 RIF/RPL testing

**FIGURE 1:**
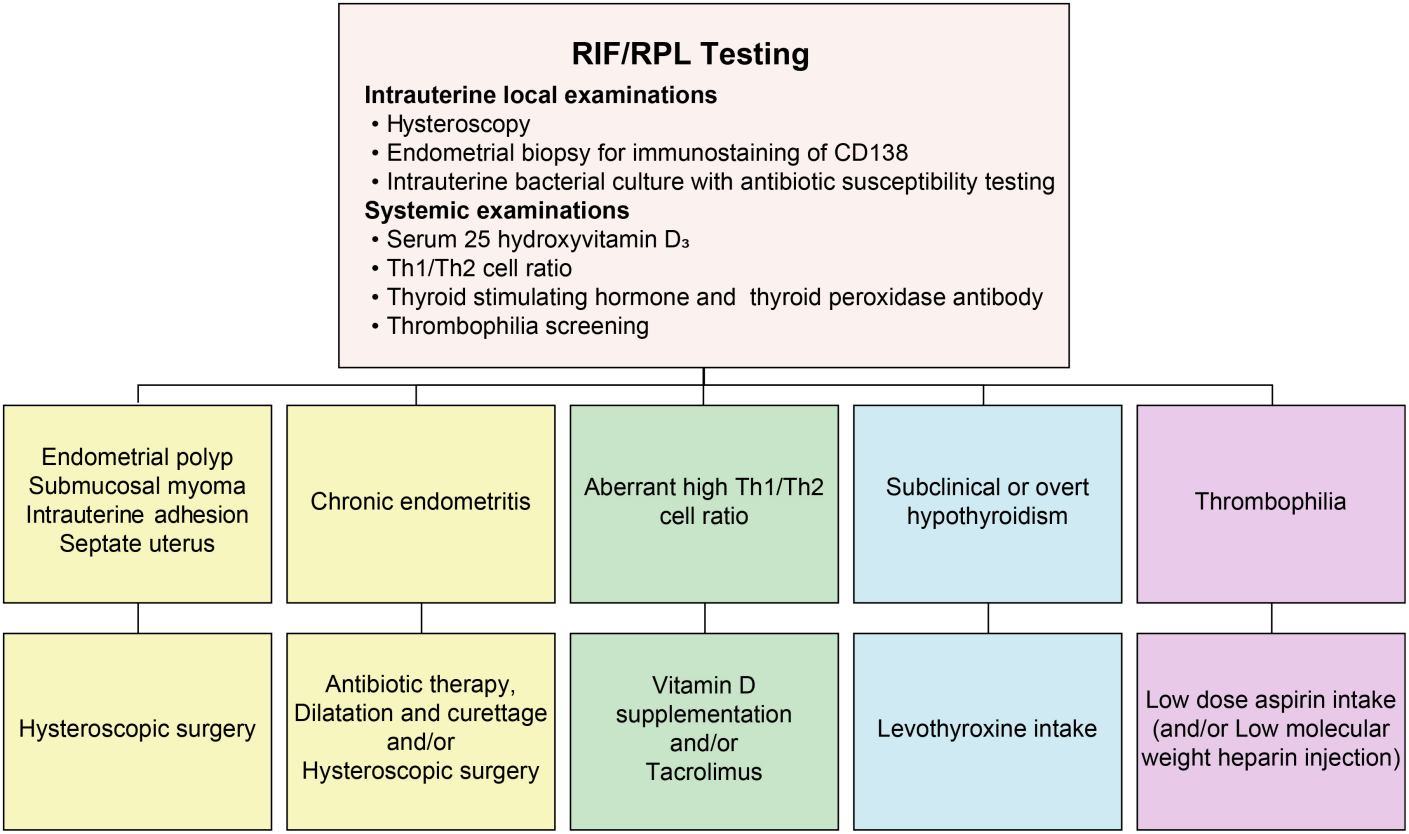
Protocol of the OPTIMUM treatment strategy. Recurrent implantation failure (RIF)/recurrent pregnancy loss (RPL) testing includes a hysteroscopy, endometrial biopsy for CD138 immunostaining and bacterial culture, and blood testing for 25‐hydroxyvitamin D_3_, IFN‐γ, and IL‐4‐producing helper T cells (Th1 and Th2 cells), thyroid function, and thrombophilia. Intrauterine abnormalities are treated through hysteroscopic surgery, chronic endometritis with antibiotics, dilatation and curettage, and/or hysteroscopic surgery; high Th1/Th2 cell ratios through vitamin D supplementation and/or tacrolimus; hypothyroidism through levothyroxine; and thrombophilia through low‐dose aspirin. Tacrolimus and aspirin use are started 1–2 days before and 5 days after blastocyst transfer, respectively, in women with RIF. In women with RPL, both are started after a positive pregnancy test. Figure in Kuroda et al. Reprod Med Biol. 2021 was modified.

The design of the OPTIMUM is presented in Figure 1. Accordingly, the OPTIMUM consists of the following RIF/RPL testing procedures: local examinations for intrauterine circumstances including hysteroscopic examination; endometrial biopsy for CD138 immunohistochemistry staining and intrauterine bacterial culture with antibiotic susceptibility testing; systemic immunological testing including assays for serum levels of 25‐hydroxyvitamin D_3_ (25OHVD) and interferon (IFN)‐γ and interleukin (IL)‐4‐producing helper T (Th) cells (Th1 and Th2 cells); thyroid function examination for thyroid‐stimulating hormone (TSH) levels and thyroid peroxidase antibody (TPOAb); and thrombophilia screening including lupus anticoagulant, anticardiolipin antibody, anti‐β2‐GP1 antibody, protein C and S activities, and factor XII.

### 2.2 Treatment based on diagnostic findings

**FIGURE 2:**
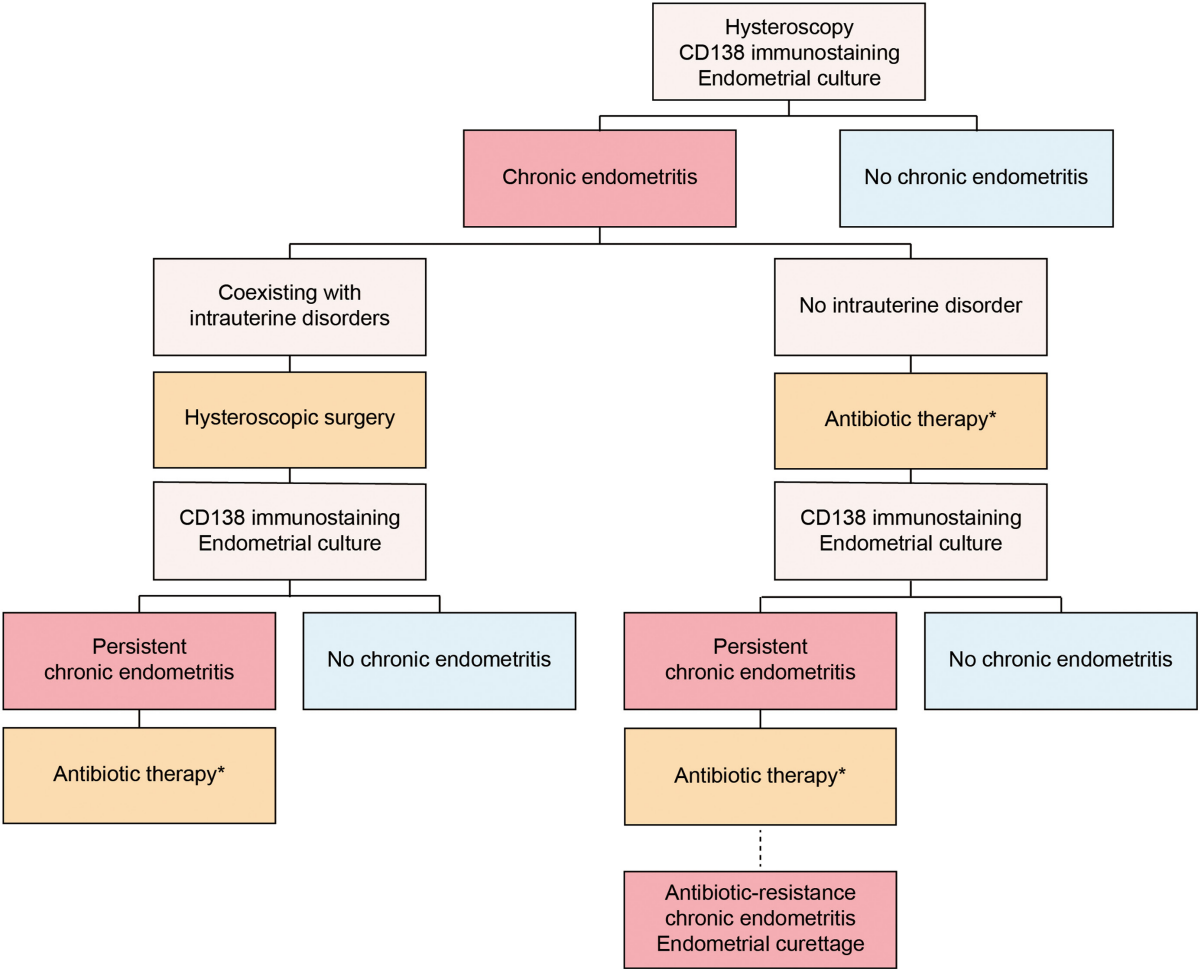
Treatment protocol for chronic endometritis. Upon detecting organic lesions via hysteroscopy, hysteroscopic surgery is performed initially. After confirming a diagnosis of chronic endometritis (CE) based on the specimen, CD138 immunostaining and bacterial culturing tests are repeated without antibiotic use in the subsequent menstruation cycle after surgery. When CE is detected in patients without intrauterine lesions, oral antibiotics for 2 weeks are administered. After two or more cycles of antibiotic use, if CE persists, gentle endometrial curettage is performed. Figure from Kuroda et al. Reprod Med Biol. 2023. *Oral bacterium‐sensitive antibiotics or doxycycline are administered, based on the results of endometrial bacterial culture tests with or without specific bacteria except for *Lactobacillus* spp. or *Bifidobacterium* spp., respectively. If CE persisted with or without specific bacteria, bacterium‐sensitive antibiotics or ciprofloxacin and metronidazole are used as second‐line therapy.

The treatment protocol for intrauterine abnormalities is summarized in Figure 2. After discovering organic lesions, such as endometrial polyps and submucosal myomas, on hysteroscopic examination, hysteroscopic surgery is initially performed. After the surgery, the collected specimen tissues are divided into two groups, after which endometrial CD138 immunostaining and bacterial culture tests are performed. The specimens are then stained using anti‐CD138 antibodies to determine the number of CD138‐positive cells. The presence of ≥5 CD138‐positive plasma cells in 10 non‐overlapping random stromal areas at 400‐fold magnification indicates a diagnosis of chronic endometritis (CE) based on our studies. Most cases of CE with intrauterine abnormalities can be cured through hysteroscopic surgery without antibiotic use.^19^, ^20^ Therefore, endometrial CD138 immunostaining and bacterial culturing tests are repeated without unnecessary antibiotic therapy during the luteal phase in the subsequent menstruation cycle after surgery. Patients without intrauterine disorders who are diagnosed with CE are given oral bacterium‐sensitive antibiotics for 2 weeks or doxycycline 100 mg twice a day for 2 weeks based on the results of endometrial bacterial culture tests with or without specific bacteria except for *Lactobacillus* spp. or *Bifidobacterium* spp. As a second‐line therapy, bacterium‐sensitive antibiotics are administered for 2 weeks or ciprofloxacin 200 mg and metronidazole 250 mg are given twice daily for 2 weeks, based on the results of endometrial bacterial culturing tests if CE persists. If CE remains even after two or more cycles of antibiotic use, gentle endometrial curettage is performed for artificial removal of the inflammatory endometrium.^21^ Our previous report showed that endometrial curettage using a blunt uterine curette without applying any force for antibiotic‐resistant CE markedly reduced the number of CD138‐positive cells, thereby improving pregnancy rates irrespective of CE persistence.^21^ Furthermore, 4.7% of those with antibiotic‐resistant CE who underwent endometrial curettage were found to have endometrial hyperplasia or cancer; therefore, removal of inflammatory endometrium can also serve as an important screening tool for endometrial cancer.

**FIGURE 3:**
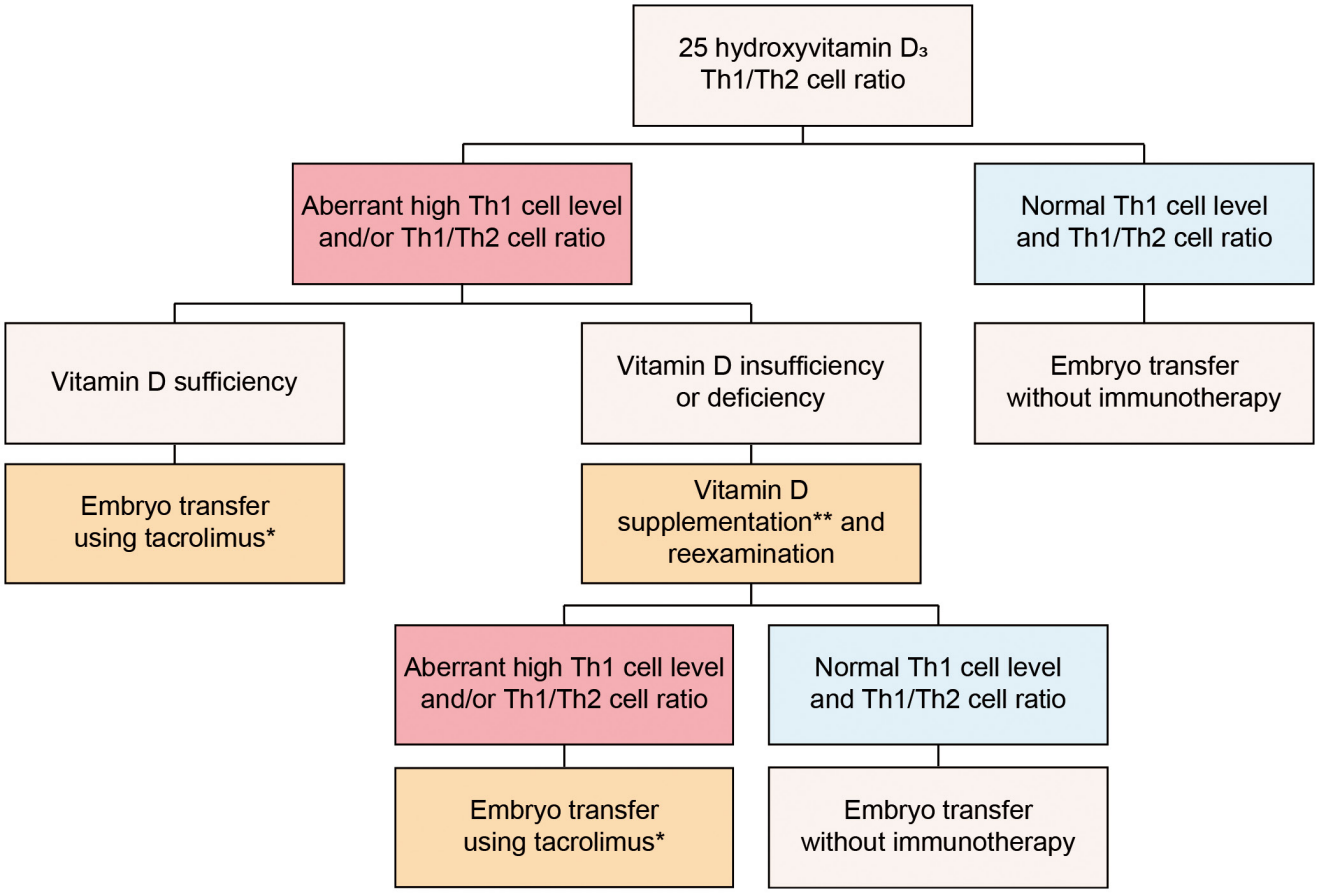
Management of maternal Th1/Th2 cell balance. Serum 25‐hydroxyvitamin D_3_ (25OHVD), Th1 (IFN‐γ), and Th2 (IL‐4) cell levels are selected for immunological testing. After confirming aberrantly high Th1 cell levels (>28.8) or Th1/Th2 cell ratios (>11.8) and low 25OHVD levels (30 ng/mL), immunological testing is repeated after ≥2 months of vitamin D supplementation at 25–50 μg/day. After supplementation, Th1/Th2 cell ratios normalized in a portion of the women, although the remaining women were treated using tacrolimus. Women with high Th1/Th2 cell ratios were treated with 2–4 mg/day of tacrolimus from 1 to 2 days before the day of embryo transfer for RIF and the day of positive pregnancy test for RPL. *Women with Th1/Th2 cell ratios of 11.8–18.9 and ≥19.0 are treated with 2 and 3 mg of tacrolimus daily, respectively. In women with Th1 cell levels of ≥28.8, the dosage of tacrolimus is increased by an additional 1 mg. **Vitamin D supplementation is provided at 25 or 50 μg daily for patients with 25OHVD levels ≥20 and <30 or <20 ng/mL, respectively.

Figure 3 depicts the management of maternal immune tolerance. Serum 25OHVD, IFN‐γ, and IL‐4 are selected for immunological testing. Notably, our previous report revealed that 25 μg of vitamin D replacement daily for 3 months promoted sufficient 25OHVD levels (≥30 ng/mL) in only half of the patients with vitamin D deficiency or insufficiency (<30 ng/mL).^22^ Therefore, vitamin D supplementation is usually provided at 25 or 50 μg daily for those with 25OHVD levels of 20–29.9 or <20 ng/mL, respectively. Data from normal fertile women and a previous trial have shown that Th1 cell levels and Th1/Th2 cell ratios of ≥28.8 and ≥11.8 should be considered aberrantly high, respectively.^23^, ^24^ Given that vitamin D replacement up to 30 ng/mL can suppress aberrantly high Th1 cell levels and Th1/Th2 cell ratios,^22^ serum levels of 25OHVD and Th1 and Th2 cells need to be reexamined after >2 months of vitamin D supplementation in the women with elevated Th1 cell levels or Th1/Th2 cell ratios and low 25OHVD levels. Our previous OPTIMUM trials found that 80%–90% of women had reduced Th1/Th2 cell ratios, half of whom were able to normalize their levels.^16^, ^17^ The remaining women with uncontrolled high Th1/Th2 cell ratios after supplementation are treated using an immunosuppressive drug, tacrolimus, as described previously.^24^, ^25^ Women with Th1/Th2 cell ratios of 11.8–18.9 and ≥19.0 are treated with 2 and 3 mg of tacrolimus daily, respectively, starting at 1 day before embryo transfer for RIF and on the day of a positive pregnancy test (4–5 weeks of gestation) for RPL. In women with aberrantly elevated Th1 cell levels (i.e., ≥28.8), the dosage of tacrolimus is increased by a further 1 mg.^24^

Regarding thyroid function, the TSH levels of 2.5–4.23 μIU/mL with TPOAb positivity or TSH >4.23 μIU/mL has been considered the treatment threshold for thyroid abnormalities using levothyroxine in women. All women receive levothyroxine therapy until their TSH levels drop to <2.5 μIU/mL and continued until live birth.

Those who fall outside of the normal range on thrombophilia screening are treated with low‐dose aspirin (81–100 mg) starting at 5 days after embryo transfer for RIF and on the day of a positive pregnancy test for RPL. Although low‐molecular‐weight heparin can also be administered to women diagnosed with antiphospholipid syndrome (APS), only one subject had received such treatment in all three OPTIMUM trials.

### 2.3 Medical interview and the modification of lifestyle

Various lifestyle habits are involved in reducing female fecundity. Lifestyle factors that increase the risks of pregnancy loss by 1.5‐ to 2‐fold include maternal and/or paternal smoking of >10–20 cigarettes/day,^26^, ^27^ caffeine intake of >2–3 cups of coffee/day,^28^ drinking >2 alcohol beverages/week,^29^ and obesity with a BMI of >30.^30^ Despite the lack of evidence on whether lifestyle modifications improve implantation rate, the ESHRE guideline for RIF recommends the evaluation and modification of lifestyle habits, such as cessation of smoking, caffeine intake, and alcohol consumption, as well as engaging in exercise and maintaining a healthy diet to combat obesity.^12^ Although the ability of supplementation to improve the fecundity of females with vitamin D deficiency still remains controversial, the ESHRE guideline for RPL states that vitamin D supplementation is safe at dosages of up to 100 μg/day and can be considered as a preventive measure against obstetrical and fetal complications.^14^ Maternal psychological stress has also been associated with progressive risk for the onset of pregnancy loss.^31^, ^32^ Therefore, counseling has been recommended to reduce their stress and anxiety as necessary.

### 2.4 Treatment for patients without risk factors

Patients with RIF/RPL who do not exhibit any risk factors for implantation failure and pregnancy loss, except for vitamin D insufficiency, may not conceive accidentally after multiple reproductive failures due to embryos or fetuses with chromosomal abnormalities. Therefore, although additional treatments may not be needed, chromosomal analysis of a couple and preimplantation genetic testing can be considered.

In women with unexplained RPL, we have recommended progesterone supplementation until 12 weeks of gestation considering that impairment of progesterone secretion during the luteal phase has been linked to miscarriage.^33^ In addition, unexplained RPL has been associated with perturbed decidualization of the endometrium,^1^, ^34^ whereas delayed timing of embryo implantation has been found to increase the risk of miscarriage.^35^ Therefore, luteal phase support with progesterone treatment could potentially prevent spontaneous abortion in the patients with unexplained RPL as previous trials.^36^, ^37^

### 2.5 Differences in the OPTIMUM treatment strategies for RIF and RPL

Table 1 summarizes the differences in the OPTIMUM treatment strategies for RIF and RPL. Accordingly, the timing of tacrolimus and low‐dose aspirin initiation intake can differ depending on whether the patient has RIF and RPL. In women with RIF, tacrolimus is started around 1–2 days before embryo transfer to support embryo implantation, whereas aspirin intake is started 5 days after blastocyst transfer given its anti‐inflammatory effects, which may inhibit embryo implantation. Among women with RPL without a history of implantation failure, both tacrolimus and aspirin are started after positive pregnancy test to prevent miscarriage. Except for the timing of aspirin and tacrolimus initiation and treatments for women without risk factors, the OPTIMUM protocols for the treatment of RIF and RPL are practically the same.

**TABLE 1:** Differences of the OPTIMUM treatment strategy for repeated implantation failure and recurrent pregnancy loss.

	RIF	RPL
Start timing of tacrolimus intake	1–2 days before blastocyst transfer	After positive pregnancy test
Start timing of low‐dose aspirin intake	5 days after blastocyst transfer	After positive pregnancy test
Treatment for women without risk factors	No additional treatment Chromosomal analysis of a couple and PGT	Progesterone treatment until 12 weeks of gestation Chromosomal analysis of a couple and PGT

Abbreviations: PGT, preimplantation genetic testing; RIF, recurrent implantation failure; RPL, recurrent pregnancy loss.

## 3 THERAPEUTIC EFFECTS OF THE OPTIMUM TREATMENT STRATEGY

### 3.1 Pregnancy outcomes of the first embryo transfer after the OPTIMUM in women with RIF

**FIGURE 4:**
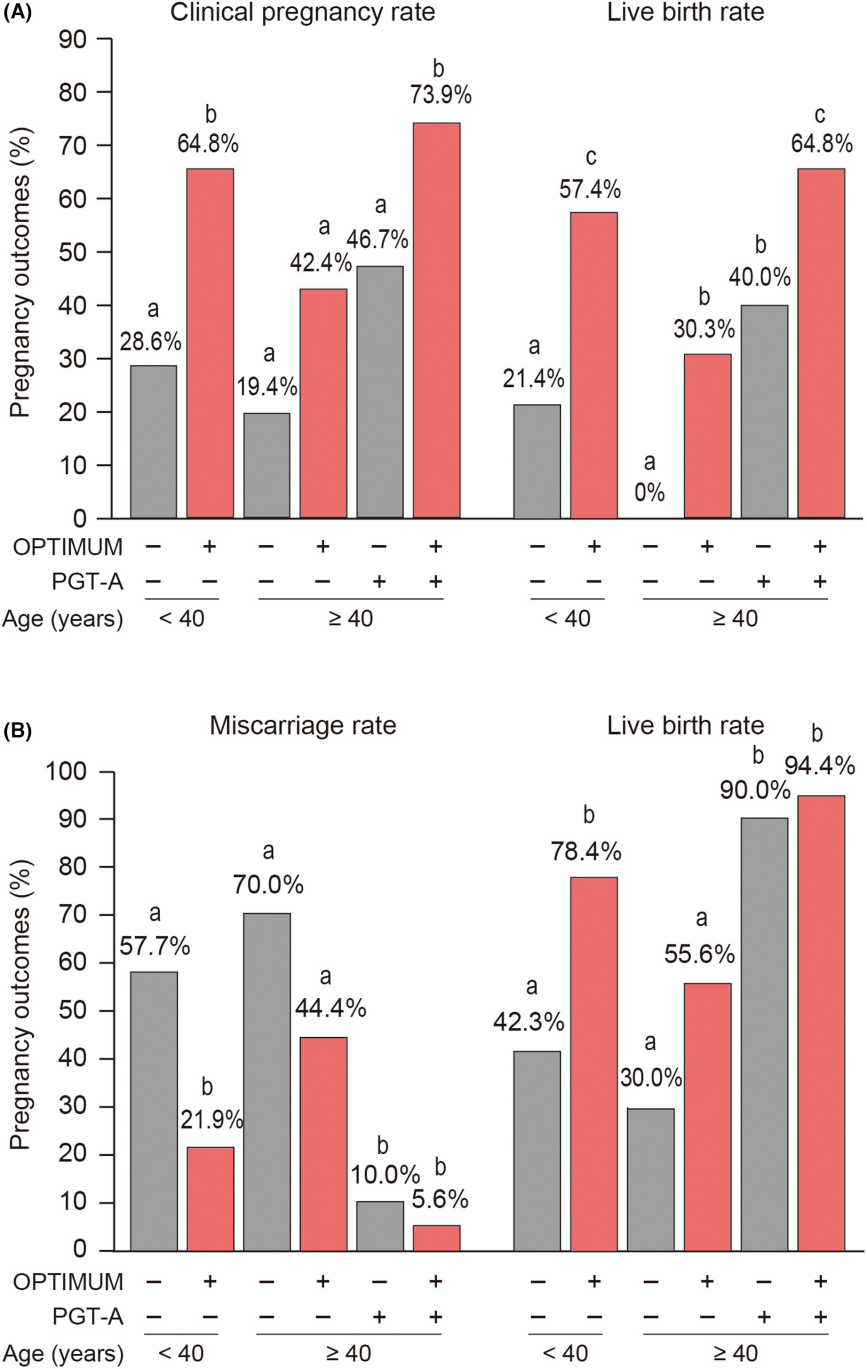
Pregnancy outcomes in previous OPTIMUM trials. (A) Pregnancy outcomes of the first embryo transfer in the OPTIMUM for recurrent implantation failure (RIF). Among women aged <40 years, 3.6% of those with RIF conceived spontaneously after the OPTIMUM. In the remaining subjects with and without the OPTIMUM, first embryo transfer produced clinical pregnancy rates of 64.8% and 28.6%, respectively (*p* = 0.005). Among women aged ≥40 years, no significant differences in clinical pregnancy rates were observed between the control, OPTIMUM, and PGT‐A groups. However, women receiving a combination of the OPTIMUM and PGT‐A had the highest pregnancy and live birth rates between the four groups (both *p* < 0.001). (B) Pregnancy outcomes of the first pregnancy after the OPTIMUM for recurrent pregnancy loss. Among women aged <40 years, those in the OPTIMUM group had significantly lower miscarriage rates than did those in the control group (21.9% and 57.7%, respectively; *p* = 0.002). Among women aged ≥40 years, those in the control and OPTIMUM groups showed no significant differences in miscarriage rates. However, when euploid blastocyst transfer was performed, miscarriage rates in those who did and did not receive the OPTIMUM were extremely low and showed no significant difference. The graphs were created based on Kuroda et al. Am J Reprod Immunol. 2021; Kuroda et al. Reprod Med Biol. 2021; and Kuroda et al. Reprod Med Biol. 2023.

Pregnancy outcomes of the first embryo transfer in two studies using the OPTIMUM for RIF were reanalyzed (Figure 4A).^16^, ^18^ Clinical pregnancy was diagnosed by confirming an intrauterine gestational sac using transvaginal ultrasound. Miscarriage was defined as a loss of clinical pregnancy, not a biochemical pregnancy. Accordingly, among women aged <40 years, 3.6% of those who experienced multiple implantation failure cycles conceived spontaneously. In the remaining subjects who did and did not receive the OPTIMUM, the first embryo transfer produced clinical pregnancy rates of 64.8% and 28.6% (*p* = 0.005) and live birth rates of 57.4% and 21.4% (*p* = 0.003), respectively. Among women aged ≥40 years, no significant differences in clinical pregnancy rates were observed between the control group and the OPTIMUM alone or PGT‐A group alone (19.4%, 42.4%, and 46.7%, respectively); however, women who received a combination of the OPTIMUM and PGT‐A had the highest pregnancy rate among four groups (73.9%, *p* < 0.001). Those who received a combination of the OPTIMUM and PGT‐A also had the highest live birth rate at 64.8% after single euploid blastocyst transfer (*p* < 0.001).

Notably, our analysis on pregnancy outcomes according to age after single euploid blastocyst transfer found that among women aged <42 years, those in the OPTIMUM group had a higher clinical pregnancy rate than did those in the control group; however, no significant differences were recognized among women aged ≥42 years.^18^

### 3.2 Outcomes of the first pregnancy after the OPTIMUM in women with RPL

After reanalyzing the miscarriage and live birth rates at first pregnancy in the two studies that used OPTIMUM for RPL (Figure 4B),^17^, ^18^ we found that among women aged <40 years, those in the OPTIMUM group had a significantly lower miscarriage rate at first pregnancy than did the control group (21.9% and 57.7%, respectively; *p* = 0.002). Meanwhile, among women aged ≥40 years, no significant differences in miscarriage and live birth rates were noted between the control and OPTIMUM groups (miscarriage rates of 70.0% and 44.4% and live birth rates of 30.0% and 55.6%, respectively; *p* = 0.095). Among advanced age women with RPL who underwent a euploid blastocyst transfer, those with and without the OPTIMUM had extremely low miscarriage rates at their first pregnancy, with no significant difference between them (5.6% and 10.0%, respectively; *p* = 0.530).

### 3.3 Factors predicting the success/failure of the OPTIMUM treatment strategy

In three OPTIMUM studies,^16^, ^17^, ^18^ predictive factors for live birth or risk factors for implantation failure or miscarriage were analyzed using multivariable logistic regression analyses (Table 2). Univariate analysis in the first trial on women with RIF showed that younger age and treatments for thrombophilia increased live birth rates after the OPTIMUM.^16^ Multivariate analysis identified thrombophilia as a predictive factor for successful OPTIMUM treatment (odds ratio [OR] = 6.38, 95% confidence interval [CI] = 1.24–32.86).

**TABLE 2:** Predictive factors for success of the OPTIMUM treatment strategy.

The subjects of the study			Main outcomes	Success group	Failure group	Univariate analysis	Multivariate analysis	References
RIF/RPL	Age (years)	PGT‐A						
RIF	25–44	No	Pregnancy outcomes within two blastocyst transfer cycles	Live birth	Implantation failure or miscarriage	Young age Thrombophilia	Thrombophilia	Kuroda et al.^16^
RPL	26–43	No	First pregnancy after OPTIMUM treatment strategy	Live birth	Miscarriage	Young age Non‐infertility	Non‐infertility	Kuroda et al.^17^
			Pregnancy outcomes within two pregnancies	Live birth	No pregnancy or miscarriage	Young age Non‐infertility High AMH level	Young age ART treatment	
RIF and RPL	40–46	Yes	Pregnancy outcomes after single euploid blastocyst transfer	Live birth	Implantation failure or miscarriage	Good‐quality embryo	No factor	Kuroda et al.^18^

*Note*: Good‐quality embryos include morphologically good blastocysts except for grade C in both the inner cell mass and the trophectoderm of the Gardner classification and 5 days of culture time from conventional IVF or intracytoplasmic sperm injection to expanded blastocysts.

Abbreviations: PGT‐A, preimplantation genetic testing for aneuploidy; RIF, recurrent implantation failure; RPL, recurrent pregnancy loss.

Univariate analysis in the OPTIMUM study for RPL demonstrated that advanced age and infertility were risk factors for pregnancy loss at first pregnancy (OR = 8.77, 95% CI = 0.96–80.45), whereas multivariate analysis identified only infertility as an independent risk factor.^17^ Among the 113 women who underwent the OPTIMUM in the mentioned study, 70 achieved live birth and 30 experienced miscarriages within two pregnancies, whereas 13 did not conceive after the OPTIMUM. Therefore, we compared 70 women who had a successful live birth and 43 with no pregnancy and miscarriages and analyzed the risk factors for unsuccessful live birth after the OPTIMUM. Accordingly, univariate analysis identified advanced age, infertility, and low AMH level as risk factors for unsuccessful live birth, whereas multivariate analysis found advanced age (OR = 1.22, 95% CI = 1.04–1.43) and infertility (OR = 3.85, 95% CI = 0.96–15.45) as independent factors for unsuccessful live birth. ART treatment was also identified as a predictive factor for successful live birth after the OPTIMUM (OR = 0.19, 95% CI = 0.05–0.73). Therefore, the risk factors for unsuccessful live birth after the OPTIMUM included advanced age, infertility, diminished ovarian reserve, and non‐ART treatment in patients with RPL.

In the third trial, which employed a combination treatment with the OPTIMUM and single euploid blastocyst transfer, univariate analysis found morphologically poor blastocysts and longer culture time until expanded blastocyst (6 days culture) as the risk factors for unsuccessful childbirth; however, multivariate analysis could not identify any predictive factors.^18^ None of the studies detected risk factors for implantation failure and pregnancy loss, including thyroid dysfunction, thrombophilia, aberrantly high Th1/Th2 cell ratio, and intrauterine abnormalities, which were treated in the OPTIMUM.

## 4 DISCUSSION

### 4.1 Indication of the OPTIMUM treatment strategy

The OPTIMUM treatment strategy has been the first combination protocol for the treatment of both RIF and RPL worldwide. The OPTIMUM can identify most of the non‐embryonic risk factors for reproductive failures through RIF/RPL testing and treat the detected abnormalities. Our trials have shown that the OPTIMUM provides beneficial effects in young women with a history of RIF and/or RPL^16^, ^17^ but did not contribute to successful pregnancy and childbearing among advanced age women with RIF and/or RPL. Female aging is strongly associated with increased risk for embryonic chromosomal abnormalities and pregnancy loss.^38^, ^39^ Among women aged 42 years or more, those who did and did not receive the OPTIMUM showed no significant difference in clinical pregnancy rates after euploid embryo transfer.^18^ In addition, euploid blastocyst transfer promoted very low miscarriage rates in advanced age women who did and did not receive the OPTIMUM.^18^ Therefore, preventing implantation failure and pregnancy loss through testing and treatment of non‐embryonic factors for pregnancy loss in advanced age women remains difficult.

**FIGURE 5:**
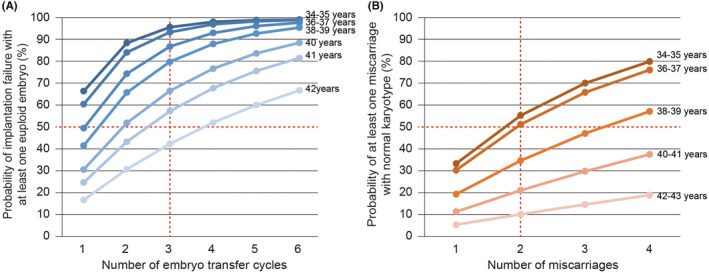
Probability of reproductive failure without embryonic or fetal chromosomal abnormalities. (A) Probability of previous implantation failures including at least one euploid embryo. The graph was created based on the PGT‐A data from the United States by Franasiak JM, et al. Fertil Steril. 2014. Women <42 years old who experienced three or more implantation failures had a ≥50% probability that transferred embryos included at least one euploid embryo. (B) Probability of previous miscarriages including at least one pregnancy with normal karyotype. The graph was created based on data for products of conception after spontaneous abortion by Segawa T, et al. Reprod Biomed Online. 2017. Women 37 years or younger had a 50% probability for two miscarriages including pregnancy with normal karyotype.

According to the PGT‐A data in the United States,^39^ euploid embryo detection rates decrease with age such that only around 50%, 42%, 31%, 25%, and 17% of women aged 35–39, 40, 41, 42, and 43 years develop a euploid embryo, respectively. Based on a previous report in cytogenic analysis of the products of conception,^6^ around 33%, 30%, 19%, 11%, and 5% of women aged 34–35, 36–37, 38–39, 40–41, and 42–43 years who had experienced a spontaneous abortion had a normal karyotype, respectively. Figure 5 summarizes the predictive rates of embryo transfer cycles including euploid embryos and miscarriages including normal karyotype. In all our OPTIMUM trials, RIF had been defined as implantation failures after three or more embryo transfer cycles using morphologically good blastocysts, whereas RPL had been defined as two or more consecutive miscarriages. Among women <42 years old who experienced three or more implantation failures, the probability that transferred embryos included euploid embryos was ≥50% (Figure 5A). Meanwhile, among women 37 years or younger, the probability of two miscarriages including a normal karyotype reaches 50% (Figure 5B). Therefore, embryo implantation failures in women aged ≥42 years with RIF or miscarriages in those aged ≥38 years with RPL can be primarily attributed to chromosomal abnormalities. Despite treating the identified risk factors, prevention of implantation failure and miscarriage in advanced age women would still remain difficult, indicating the need for PGT‐A.

### 4.2 Risk or protective factors for the OPTIMUM treatment strategy

APS in thrombophilia disorders is a well‐known risk factor for RPL. Patients with a history of RIF have a relatively high prevalence of thrombophilia.^40^, ^41^ In fact, our study on the OPTIMUM showed that 20%–30% of patients with RIF had thrombophilia,^16^, ^18^ suggesting that treatment of thrombophilia is a predictive factor for RIF.^16^ Therefore, assessment and treatment of thrombophilia are important for pregnancy and childbearing in women with not only RPL but also RIF. However, no evidence has shown that thrombophilia is a risk factor for implantation failure. In fact, a randomized controlled trial revealed that treatment using low‐dose aspirin and heparin did not improve clinical pregnancy rates in patients with RIF who had antiphospholipid or antinuclear antibodies.^42^ A systematic review demonstrated that the use of heparin in women with RIF improved live birth rates and decreased miscarriage rates but had no effect on clinical pregnancy rates.^43^ Low‐dose aspirin and heparin are usually administered after confirming pregnancy for the prevention of miscarriage. Thus, starting aspirin use before the timing of embryo implantation is unnecessary given that embryo implantation is not supported by aspirin.^44^, ^45^, ^46^ In the OPTIMUM protocol for RIF, aspirin treatment is started 5 days after blastocyst transfer, which falls within post‐implantation period.

The risk factors for failure of the OPTIMUM in women with RPL included infertility and non‐ART treatment.^17^ Regarding infertility, women with infertility had a significantly higher pregnancy loss than did those without infertility (39.7% and 4.5%; *p* = 0.001).^17^ During infertility treatment, successful pregnancy and fetal loss induce intense psychological stress,^47^ which causes an immunological response via an increase in the Th1/Th2 cell balance with a bias toward Th1.^48^ Furthermore, repeated exposure to semiallogeneic embryos or fetuses may induce systemic immune rejection. In fact, studies have shown that women with a history of RIF or RPL exhibited significantly higher Th1 cell levels and Th1/Th2 cell ratios than in those with general infertility.^23^ Thus, women with both infertility and RPL may be a distinct population who experience exceptional difficulty in having children.

Nevertheless, it is important to promote pregnancy and childbirth as soon as possible before their miscarriage rates increase with female aging and immune rejection. ART treatment is also needed to induce pregnancy without delay in advanced age women with a history of RPL who have even conceived spontaneously. Evidence shows that the cumulative live birth rates over 5 years in RPL women aged 30–34, 35–39, and ≥40 years are approximately 70%, 60%, and only 40%, respectively.^49^ Therefore, patients aged ≥40 years with RPL require ART treatment (including PGT‐A as an additional option) to achieve successful childbirth. ART treatment should also be considered for young women with low AMH levels.

### 4.3 Evidence‐based treatment strategies for RIF and RPL

Although RIF and RPL are multifactorial diseases, no combination treatment protocols based on diagnostic findings have been reported. The OPTIMUM targets risk factors for implantation failure and pregnancy loss, including thyroid function, thrombophilia, immune status, and intrauterine circumstance. The ESHRE guideline for RIF states that assessments of thyroid function, APS, intrauterine environment, lifestyle factors, karyotyping, and progesterone levels can be recommended or considered.^12^ Meanwhile, the guideline for RPL recommends or conditional recommends assessments of thyroid function, APS, uterine disorders, and lifestyle factors in couples.^14^, ^15^ Although the OPTIMUM covers thyroid, thrombophilia, uterine, and lifestyle factors, available guidelines do not recommend testing for immune factors due to lack of evidence. The guideline for RPL recommends the use of repeated and high doses of intravenous immunoglobulin as immunotherapy for four or more instances of unexplained RPL.^15^ While intravenous immunoglobulin can reduce peripheral cytotoxic NK cells and suppress aberrant high Th1/Th2 cell ratio,^50^ this treatment is extremely expensive and has some serious side effects.^51^ Vitamin D has also been shown to suppress elevated Th1/Th2 cell ratios and NK cell activity.^22^, ^52^ In fact, vitamin D insufficiency/deficiency has been associated with infertility and complications during pregnancy, including pregnancy loss, preeclampsia, and gestational diabetes mellitus.^53^, ^54^, ^55^, ^56^ Furthermore, assessment of vitamin D levels and subsequent supplementation is low cost and very safe. Despite a number of meta‐analyses showing the therapeutic effects of vitamin D supplementation on pregnancy loss and pre‐eclampsia,^57^, ^58^, ^59^ the assessment of vitamin D levels and treatment of vitamin D insufficiency/deficiency are not recommended in the ESHRE guideline for RIF.^12^ More evidence on the immunomodulatory effects of vitamin D use in preconception care is needed for patients who suffer from RIF and RPL.

### 4.4 Cost‐effectiveness

Most women with a history of RIF or RPL feel physically and psychologically burdened given the exorbitant costs associated with testing and treatments required for childbearing.^60^, ^61^ Some women develop social problems due to long‐term infertility treatments and repeated pregnancy losses and stillbirth and often suffer from mental depression, anxiety disorders, and insomnia.^62^, ^63^, ^64^ Therefore, a cost‐effective protocol with inexpensive testing and treatment that produces good pregnancy outcomes is imperative. Considering that the RIF/RPL testing protocol in our OPTIMUM treatment strategy does not include expensive examinations, such as ERA and endometrial microbiome testing, the entire treatment would cost around 50 000–60 000 yen ($US 340–400),^17^ with the Japanese medical insurance covering a portion of these tests in women with a history of two or more consecutive pregnancy losses. Therefore, patients should not have to endure a significant financial burden resulting from the OPTIMUM.

### 4.5 Beneficial effects of the combination treatment protocol for RIF and RPL

In the OPTIMUM, we have targeted thyroid dysfunction, thrombophilia, aberrantly high Th1/Th2 cell ratio, and intrauterine abnormalities for the treatment of RIF and RPL. These risk factors may interact complicatedly and overlap each other. For instance, vitamin D deficiency is associated with not only high Th1/Th2 cell ratio but also APS and thyroid autoimmunity.^55^, ^65^ Furthermore, CE is involved in aberrantly high Th1 and low Th2 cell levels in local endometrium.^66^ Therefore, the combination treatment protocol may have further therapeutic effects by optimizing each of these risk factors.

Furthermore, RIF and RPL are strongly involved in psychological stress,^47^, ^60^, ^61^, ^62^, ^63^, ^64^ and treatment with placebo has been shown to relieve maternal stress and reduce the pregnancy loss rate.^67^ In our OPTIMUM trials, the control groups did not receive a placebo; therefore, the interventions in the OPTIMUM groups may improve pregnancy outcomes through the placebo effect.

## 5 CONCLUSION

Implantation failure and pregnancy loss have been associated with various risk factors. The OPTIMUM treatment strategy, which simply detects risk factors using inexpensive tests and treats them, has been the first combination protocol for both RIF and RPL worldwide. This treatment strategy can address most non‐embryonic factors and promote pregnancy and childbearing in most young patients with RIF or RPL or those undergoing euploid embryo transfer. However, the OPTIMUM is, of course, not a perfect treatment and may include unnecessary testing and treatment procedures, as well as some other examinations that further improve pregnancy outcomes, such as selection of sperm with low DNA fragmentation. The combination treatment protocol needs to be further refined and developed in the future for the couples suffering from RIF and RPL.

## FUNDING INFORMATION

There are no funders to report for this submission.

## CONFLICT OF INTEREST STATEMENT

The author has no conflicts of interest to declare relevant to this study. Human rights statement: These studies were approved by the local ethics committee of Juntendo University, Faculty of Medicine (No. 14–103) and Sugiyama Clinic (No. 18–002 and 22–008). All procedures followed were in accordance with the ethical standards of the responsible committee on human experimentation and with the Helsinki Declaration of 1964 and its later amendments. All recruited women provided written informed consent. The data that support the findings of this study are available on request from the corresponding author. The data are not publicly available due to privacy or ethical restrictions. Animal studies: This article does not contain any study with animal participants that have been performed by any of the authors.
